# Research on the Psychological Expectation Impact on Enterprise Innovation Under Mergers and Acquisitions Pressure: From the Evidence of Large Stock Dividends

**DOI:** 10.3389/fpsyg.2021.744875

**Published:** 2021-09-28

**Authors:** XueWen Kuang, ZeYu Li, He Lin

**Affiliations:** ^1^School of Economics and Management, Nanchang University, Nanchang, China; ^2^School of Business, Sichuan University, Chengdu, China

**Keywords:** stock dividends, psychological expectation, stock liquidity, enterprise innovations, M&A pressure

## Abstract

From the perspective of social psychology, takes the large stock dividends policy of Chinese listed companies as an example, based on the sample of Chinese listed companies from 2009 to 2018, this article examines the impact of psychological expectation under the mergers and acquisitions (M&A) pressure on enterprise innovation. The empirical study finds that the high dividend payout mainly increases the liquidity of the stock, which makes the company face a greater risk of hostile merger and acquisition, and thus causes the management to pay attention to the psychological preference of short-term effect, and reduces the level of enterprise R&D and innovation. Moreover, the above conclusion is still true after controlling the endogenetic problem. Further research shows that in private enterprises, enterprises with low ownership concentration and non-high-tech enterprises, large stock dividends has a more obvious inhibiting effect on the level of enterprises’ R&D and innovation. After excluding the possible of agency problem, the conclusion is still robust. This article expands the research on the influencing factors of enterprise innovation from the perspective of psychological expectation. The findings of this study provide references and inspirations for facilitating enterprise innovations by reducing short-sighted behaviors of management under increased stock liquidity.

## Introduction

Innovation is the booster of macroeconomic growth. It is inseparable from the transformation of economic development methods and the improvement of the quality of economic development. For micro-economic entities, innovation plays an important role in maintaining core competitiveness (Porter, 1992). Existing research on the factors affecting enterprise innovation has been very abundant. Factors such as industry characteristics, stakeholders, and corporate governance will all affect corporate innovation (Ferreira et al., 2012; Zhang and Xue, 2021). Innovative activities are highly risky and require a large amount of funding. The liquidity of stocks affects the trading environment and provides the possibility of affecting corporate innovation activities. Fang et al. (2014) and Wen et al. (2018) used data from the United States and China, respectively, to confirm that stock liquidity is one of the key factors affecting corporate innovation.

An interesting phenomenon in the Chinese capital market is that listed companies pay dividends in the form of bonus shares or capital accumulation. This behavior, along with stock splits and stock splits, will increase the number of stocks in circulation and may also cause higher stock liquidity. In essence, the transfer of shares is only an adjustment between different accounting items within the owner’s equity, and will not have any impact on the total value of the company. Therefore, some scholars jokingly claim that the transfer of shares is just a “plastic surgery” operation by dividing the company’s “pie” into more “small pieces” (Adaoglu and Lasfer, 2011). However, in recent years, the phenomenon of share transfers by listed companies has become more and more intense.

In order to uncover the hidden mystery of the stock swap, scholars, respectively, from the management to cater to the needs of irrational investors, insiders to reduce their holdings and catering to the needs of external investors participating in private placement (Bushman and Piotroski, 2006; Kumar, 2009; Weld et al., 2009; Chen et al., 2013; Devos et al., 2015; Birberick, 2020; Ludwig et al., 2020). In general, behavioral finance can better explain the phenomenon of share transfer. On the investor side, irrational investors often have a gambler mentality. The occurrence of share transfers makes them believe that the stock price has fallen to a reasonable price range, and that they have found high-quality “bargains.” When managers consider stock dividends, an important goal is to reduce stock prices to improve stock liquidity (Baker and Powell, 1993). When managers are rational and investors are irrational, managers will cater to the irrational preferences of investors in order to maximize their own interests. Therefore, the transfer of shares is the result of the game between investors and managers.

The transfer of shares is similar to stock splits, which means the increase in the number of shares in the capital market, the reduction of stock prices and transaction costs, which may lead to higher stock liquidity. At the same time, on the one hand, stock liquidity may promote mergers and acquisitions between enterprises, making managers face the pressure of mergers and acquisitions. Social psychology theory believes that the environmental atmosphere will affect people’s motivations, thereby affecting people’s behavioral decisions (Kerksieck et al., 2019; Wu et al., 2020). The psychological expectation of mergers and acquisitions (M&A) pressure will aggravate management’s short-sighted behavior, thereby reducing corporate innovation (Nguyen and Nguyen, 2020; Garcia-Sanchez, 2021). On the other hand, it is possible to improve corporate governance by introducing new shareholders, so that managers are willing to strengthen corporate innovation for long-term development.

Will the transfer of shares affect stock liquidity? Will the stock liquidity caused by the transfer of shares affect the level of corporate innovation? The answers to the above questions build a bridge between the transfer of shares and corporate innovation, enrich the research on corporate innovation by stock liquidity and corporate governance, and provide a new perspective. At the same time, using the theory of behavioral finance to analyze, more vividly analyze the effect of the transfer of shares on enterprise innovation. The research takes Chinese A-share listed companies from 2009 to 2018 as the research sample to investigate the impact of share-swapping on the innovation level of listed companies, explore the mechanism of share-swapping, and distinguish the impact of share-swapping on the innovation behavior of listed companies in different situations.

The results of this study demonstrate that stock dividends are negatively related to innovations of listed companies, especially private enterprises, enterprises with low equity ownership concentration and non-high-tech enterprises. This conclusion is solid even if agency induced by stock dividends are eliminated, suggesting that the effects of stock dividends on enterprise innovations are not related to agency. Further analysis revealed that increased stock liquidity plays a key role in degradation of innovation level of enterprises induced by stock dividends. Specifically, increased stock liquidity leads to increased pressure of hostile takeovers and management has to sacrifice investment for long-term development to guarantee short-term revenue. The contributions of this study are mainly in three aspects. First, this study provides an alternative perspective to clarify factors affecting enterprise innovations, though various studies in this field have been reported. Specifically, stock dividends are introduced into the analytical framework of innovations and their effects on innovations are discussed. Second, this study provides empirical evidences to thorough understanding of economic consequences of stock dividends of enterprises. Previous studies focused on motivations of stock dividends, while we investigated the economic consequences of correlation of stock dividends and enterprise innovations. Third, the working principles of stock dividends on enterprise innovations were investigated and the results verified the stock liquidity hypothesis of stock dividends, thus enriching theories about stock dividends of enterprises.

The remainder of this article is arranged as follows: The second part is literature review and research hypothesis; The third part is the method; The fourth part is the empirical results; The fifth part is the research and discussion; The sixth part is the theoretical contribution; The seventh part is the research conclusion.

## Literature Review and Hypotheses

Stock dividends are essentially stock split (direct split, which is conversion of capita reserve and indirect split, which is distribution of bonus shares) and have no influences on the total value of the company (Grinblatt et al., 1984). Nevertheless, some investors pursue listed companies distributing stock dividends, especially large stock dividends. In order to clarify this phenomenon, signal transmission theory, optimal stock price range theory, cash substitution theory and liquidity theory have been proposed. The signal transmission theory claims that replacing cash dividends by stock dividends is a signal to the investors that the management is optimistic about the future of their company (Asquith et al., 1989; Louis and Robinson, 2005). The optimal stock price range theory claims that the management aims to reduce the over-high share prices of the company to an acceptable level by stock split so that more investors are willing to invest in the company (Grinblatt et al., 1984; So and Tse, 2000). The cash substitution theory believes that listed companies tend to substitute cash dividends with stock dividends so that they have sufficient cash for external investment opportunities (Stice, 1997; Brav et al., 2005). The liquidity theory believes that reduction of share prices by distribution of stock dividends is a tradeoff of increased investor base and reduced transaction cost (Lakonishok and Lev, 1987; Brennan and Copeland, 1988).

Nevertheless, contradictory conclusions have been drawn on whether these theories can explain stock dividends in China (Huyghebaert and Wang, 2016; Hu et al., 2021). Studies of stock dividends, especially large stock dividends, of China’s listed companies reveal that the “Nominal Stock Price Illusion” hypothesis may explain the enthusiasm of investors on listed companies distributing stock dividends. By distributing stock dividends, listed companies are trying to convince irrational investors that low price stocks have a large space to rise and a small possibility to fall. In this way, these companies achieve market value management during right filling (Birru and Wang, 2016). However, consistent evidences are absent to determine whether stock dividends of China’s listed companies satisfy the liquidity theory (Chen et al., 2010). Based on a data set of up to 1,232 announcements of China’s capital market which demonstrated the signal transmission theory while no conclusions on whether the liquidity theory can explain distribution of stock dividends by listed companies had been drawn. Nguyen and Wang (2013) claimed that distribution of stock dividends by China’s listed companies satisfies the liquidity theory.

To date, various studies of factors influencing innovations have been reported. All proposals contributed to enhancement of innovations by listed companies. Fang et al. (2014) reported for the first time that stock liquidity is negatively instead of positively related to innovations of listed companies based on the data of American companies. Wen et al. (2018) reported similar conclusions based on the data of Chinese companies. In both cases, increased stock liquidity leads to increased pressure of hostile takeovers. As a result, management must keep the share price at a relatively high level and has no motivation on innovations, which are characterized by high risk and uncertainty. Distribution of stock dividends by listed companies will increase the number of stocks in circulation and reduce share prices to an acceptable level at the cost of expanded stock base. In this way, more investors are attracted and its stock liquidity is enhanced (Adaoglu and Lasfer, 2011). As a new economy in transition, China is characterized by high percentages of transactions by minority investors and low market liquidity. Compared with institutional investors, minority investors prefer stocks at low prices due to their limited financing volume. For this reason, distribution of stock dividends can reduce stock price to attract minority investors, thus increasing stock liquidity.

What are the effects of increase of stock liquidity induced by stock dividends on enterprise innovations? Herein, we propose that increase in liquidity by distribution of stock dividends will affect innovations of listed companies in two aspects. First, high stock liquidity leads to increased pressure of hostile takeovers on listed companies (Amit et al., 1989; Smith and Kim, 1994; Guo et al., 1995; Fang et al., 2014). With high stock liquidity, external potential buyers can disguise their acquisitions as ordinary transactions due to the presence of the large number of liquidity traders (Stein, 1988). As one of the most extreme examples of external pressure on management, hostile takeovers may force management to invest in routine projects with rapid and guaranteed returns for career reasons (Atanassov, 2013). Innovations, which exhibit long return cycle, high risk and uncertainty (Holmstrom, 1989) is inconsistent with the interests of the management at this stage and the willingness of management to invest in R&D is extremely low. Holmstrom (1989) found that capital markets tend to force management by pressure to focus on short-term projects and ignore innovation. Shleifer and Summers (1988) believed that the management has less rights and incentives to invest efforts in innovations compared with shareholders. When facing severe threats of hostile takeovers, the management worries that hostile buyers can share the profits brought by innovations without bearing the cost, which may cause them to be fired. Hence, the risk of hostile takeovers leads reduced incentive of innovations for the management. This conclusion has been verified by the data of both American (Fang et al., 2014) and Chinese (Wen et al., 2018) companies.

Increased stock liquidity leads to reduced transaction cost, which makes it easier for institutional investors to exit. The theory of short-sighted institutional investors claims that most institutional investors focus on short-term performances, resulting in short-term behaviors of listed companies (Porter, 1992). Ferreira et al. (2012) believed that any factors favoring short-term behaviors will directly hinder innovations. Graham et al. (2005) investigated 400 listed companies in United States and found that 78% of all CFOs would sacrifice long-term projects to meet some short-term benefits if they are facing high performance pressure. Likewise, institutional investors are also facing performance pressure and they expect invested enterprises to generate sustainable income. At the initial stage of innovation activities, the risk of innovation may cause severe fluctuations of enterprise’s performance. When the expected performances of listed companies fail to meet their requirements, institutional investors may no longer wait for potential benefits brought by innovations. In this case, reduction of transaction cost caused by high stock liquidity makes its exit easier. In order to avoid the impacts of stock price fluctuation on short-term revenues, management of listed companies tend to reduce innovation cost (Porter, 1992).

Based on the above analysis, the following hypotheses are proposed:

H1: When other conditions remain unchanged, the degree of stock transfer is negatively correlated with the level of enterprise innovation.

Property rights, as the core of the enterprise system, will affect the choices and decisions of enterprises, thus affecting the innovation behavior and innovation performance of enterprises (Marshall, 1907). This article argues that the property right nature plays a moderating role in the relationship between the transfer of shares and the innovation level of enterprises. The specific reasons are as follows: First, according to our theoretical analysis above, stock transfer improves the liquidity of enterprises and leads to increased pressure of hostile takeover of enterprises, which leads to short-sighted behavior of management. In M&A cases, most of the state-owned enterprises (SOEs) play the role of acquirers are rarely acquired by external shareholders (Florio et al., 2018). The pressure from external hostile M&A is relatively small for SOEs, leaving enough space for SOEs to carry out innovation. Private enterprises, on the other hand, if you can’t meet the requirements of investors and business performance by investors, in the case of stock liquidity increase will face a greater risk of a hostile takeover, buy inferior quality enterprise is the rule of market development and an important way to redistribute resources, under the pressure will intensify short-sighted behavior of the management of private enterprises. Second, state-owned enterprises generally have the characteristic of “single dominant share,” and the state is the major shareholder of state-owned enterprises. This natural attribute ensures the concentration of state-owned enterprises’ equity, and the stock liquidity increased by the transfer of shares has little impact on state-owned enterprises. The equity of private enterprises is highly dispersed, and the liquidity increases the pressure of hostile takeover of private enterprises, which aggravates the short-sighted behavior of the management of private enterprises. Third, according to the human resources and social security and so on six department in 2009 issued “about further standard, head of the central enterprise salary management guidance,” known state-owned enterprises strictly limit executive pay, less type performance pay of executive incentive in state-owned enterprises, more is through the political promotions and on-the-job consumption (Firth et al., 2006). while the sensitivity of executive compensation performance in private enterprises is stronger. Short-sighted behavior caused by the pressure of hostile takeover caused by liquidity can reduce the fluctuation of performance to a certain extent, which is more in line with the interests of the management of private enterprises with strong sensitivity to pay and performance.

Therefore, the following hypotheses are proposed:

H2: The negative impact of stock transfer on enterprise innovation is more significant in private enterprises.

Further, we explore the moderating effect of ownership concentration on the transfer of shares and the level of innovation. When the listed company is in the situation of high ownership concentration, the influence of stock liquidity increased by the transfer of shares on the listed company is small, and the risk of hostile takeover is reduced, and the degree of weakening of innovation level will also be reduced. When the equity concentration of listed companies is low, and the liquidity raised by stock transfer increases the pressure of hostile takeover, the major shareholders reduce the impact on short-term earnings by sacrificing long-term projects such as innovation (Stein, 1988), so as to reduce the impact on current stock price.

Therefore, we believe that:

H3: In the case of high ownership concentration, the negative impact of stock transfer on innovation level will be weakened.

Finally, innovation activities are characterized by long-term nature and risk. The innovation activities carried out by enterprises mean the change of the past and the enhancement of the uncertainty in the future. However, enterprises in different industries have different sensitivities to innovation activities (Hsu et al., 2014). According to the above analysis, we know that equity transfer significantly inhibits the innovation level of enterprises, so is there a significant difference between high-tech enterprises and non-high-tech enterprises? Innovation is a rigid demand for high-tech enterprises. The characteristics of high-tech enterprises determine that enterprises must continue to innovate if they want to occupy a favorable market position in the market. Therefore, it is theoretically expected that:

H4: In high-tech enterprises, the negative impact of stock transfer on enterprise innovation level will be weakened.

## Research Design

### Sample Selection and Data Sources

In this study, companies in A-shares of Shanghai and Shenzhen Stock Exchange that distributed stock dividends in 2009–2018 were selected as the subjects. 2009 was selected as the starting year because listed companies have been required to disclose information about their investment in R&D since 2007, while information quality of annual reports in 2007 and 2008 was relatively low. The shortage of chip in happened in 2019 has caused enormous impact for the innovation among corporates at home, not mentioning the pandemics caused by COVID-19, the majority of the company were confronted with the financial strain, showing a distinct flux in the investment in research and development between 2019, 2020 and the year forward. Based on the situation mentioned above, this article selects original source of the data and information in the span of 2009–2018 as the observation data. Additionally, data of listed companies in finance were excluded, abnormal data were excluded, data of companies with missing data were excluded, and data of ST and ^∗^ST companies were excluded. Since outliers may affect the empirical results, I trim all the firm-level variables at the 1st and 99th percentiles. The turnover rates were obtained from the RESSET Database, the R&D investments were obtained from the Wind Database, while other data were obtained from the CSMAR Database.

The model estimation in this article is mainly carried out by Stata 14. In the hypothesis testing stage, the fixed-effects unbalanced panel regression is performed, while the industry and the year are controlled, and the standard error is passed through the enterprise-level cluster. In terms of endogeneity testing, propensity score matching method, Heckman two-stage model, and double difference estimation model (DID-PSM) are used to control the endogeneity problem.

### Regression Model and Variable Definitions

The following model was established to verify the proposed hypothesis (Stein, 1988; Fang et al., 2014):


(1) $$R\,D_{i}=\beta_{0}+\beta_{1}\,S\,e\,n\,d\,\_\,r\,a\,t\,i\,o_{i}+\beta_{k}\,C\,V_{i}+Y\,e\,a\,r+I\,n\,d+\varepsilon \,i$$

where RD, which reflects innovation level of enterprises, is the dependent variable (RD = investment in R&D/revenue). Send_ratio, which reflects the transfer capacity, is the independent variable (Send_ratio = proportion of stock dividends in the current year + proportion of stock conversion in the current year). A variety of factors, such as the size of the enterprise, the time of its establishment, operating conditions, growth opportunities, equity structure, and financial status, all affect enterprise innovation. The larger the scale of the enterprise, the better the operating conditions, and the forecast of the controlling shareholder’s shareholding ratio, the higher the enterprise’s high risk tolerance for innovative activities will enable managers to maintain long-term profit goals; companies with stronger growth opportunities and lower debt levels are easier to raise funds, which guarantees the funding requirements for innovative activities. Companies with more cash flow also have no worries about funding issues. Others are control variables (see Table 1).

**TABLE 1:** Symbols, names, and definitions of key variables.

	Symbol	Name	Definition
Dependent variable	RD	Research density	Ratio of R&D investment to main business revenue in the current period
Independent variables	Send_ratio	Transfer ratio	The percentages of dividend and conversion in the current year
Control variables	Lnsize	Enterprise size	Natural logarithm of total assets at the end of the period
	LEV	Capital structure	Ratio of total liabilities to total assets at the end of the period
	ROA	Profit margin	Ratio of net profit to total assets at the end of the period
	Tobin Q	Business growth opportunities	(Market value – total liabilities)/total assets at the end of the period
	LnAge	Enterprise age	Log (current year – registration year +1)
	CFO	Free cash flow	Ratio of net cash flow from business activities to total assets
	TOP1	Proportion of shares held by the controlling shareholder	Proportion of shares held by the controlling shareholder in the current year
	Capital	Capital expenditure	The ratio of cash paid for purchase/construction of fixed assets, intangible assets and other long-term assets to total assets in the current year
	PPETA	Capital intensity	The ratio of net fixed assets to total assets in the current year
	Sales	Profitability	Natural logarithm of sales revenue in the current year
	Year Dummy	Dummy variable of year	1 if it is in this year; 0 if not
	Industry Dummy	Dummy variable of industry	1 if it is in the industry; 0 if not

To make the results convincing, the regression results of both the current period (Period *t*) and the period behind the current period (Period *t*-1) were included. In order to control their impacts, industry and year were added into the regression as dummy variables. Additionally, clustering of standard error is observed at enterprise level. β1 is expected to be significantly negative. In other words, distribution of stock dividends leads to degraded enterprise innovations.

## Empirical Results

### Descriptive Analysis

Table 2 shows the descriptive statistics of main variables. As observed, mean and standard deviation of research density (RD) are 0.0486 and 0.0446, respectively, demonstrating large differences in R&D investments by listed companies. The median and standard deviation of ratio of stock dividends (Send_ratio) were 0.6 and 0.1, respectively, demonstrating high ratio of stock dividends of listed companies and large deviations in ratios of stock dividends of different companies. Mean and standard deviation of the shareholdings of the largest shareholder (TOP1) are 0.347 and 0.136, respectively, suggesting large differences in equity ownership concentration of different companies.

**TABLE 2:** Descriptive analysis.

Variable	*N*	Mean	Median	SD	Min	Max	Skewness	Kurtosis
RD	4313	0.0486	0.0374	0.0446	0.0004	0.257	2.3447	9.8693
Send_ratio	4313	0.731	0.600	0.429	0.100	2.100	0.9947	3.9705
Lnsize	4313	21.57	21.38	1.041	19.87	24.87	0.8618	3.4970
Lev	4313	0.323	0.298	0.182	0.0336	0.777	0.4758	2.3898
ROA	4313	0.0666	0.0608	0.0408	−0.0030	0.208	1.012	4.3568
Tobin Q	4313	3.155	2.561	2.234	0.346	12.10	1.6358	6.1541
LnAge	4313	2.570	2.639	0.451	1.099	3.332	−0.9242	3.9668
CFO	4313	0.0450	0.0445	0.0696	−0.155	0.234	−0.0576	3.5659
TOP1	4313	0.347	0.336	0.136	0.0920	0.700	0.3972	2.6242
Capital	4313	0.0632	0.0483	0.0543	0.0016	0.268	1.4956	5.3272
PPETA	4313	0.175	0.151	0.123	0.0041	0.556	0.9074	3.4496
Sales	4313	20.90	20.75	1.224	18.67	24.68	0.6972	3.3478

Table 3 shows the correlation analysis results of the main variables of the full sample, the Pearson correlation coefficient shows: The R&D density (RD) and the share transfer ratio (Send_ratio) are significantly positively correlated, and the significance level is above 5%. The correlation coefficient between each variable is less than 0.5, indicating that the problem of collinearity is not big.

**TABLE 3:** Correlation analysis.

	RD	Send_ratio	Lnsize	Lev	ROA	Tobin Q	LnAge	CFO	TOP1	Capital	PPETA	Sales
RD	1											
Send_ratio	0.030**	1										
Lnsize	−0.243***	−0.046***	1									
Lev	−0.317***	−0.059***	0.588***	1								
ROA	0.051***	−0.066***	−0.089***	−0.360***	1							
Tobin Q	0.291***	0.213***	−0.410***	−0.422***	0.421***	1						
LnAge	−0.102***	−0.073***	0.246***	0.213***	−0.029*	−0.085***	1					
CFO	0.0160	−0.080***	0.068***	−0.139***	0.482***	0.203***	0.079***	1				
TOP1	−0.176***	–0.0220	0.071***	0.057***	0.059***	–0.0230	−0.057***	0.073***	1			
Capital	−0.054***	0.0150	−0.051***	–0.00700	0.037**	−0.037**	−0.154***	0.094***	0.049***	1		
PPETA	−0.256***	−0.058***	0.130***	0.154***	−0.080***	−0.168***	0.073***	0.239***	0.094***	0.360***	1	
Sales	−0.367***	−0.102***	0.894***	0.620***	0.0180	−0.393***	0.239***	0.127***	0.112***	−0.037**	0.184***	1

***, **, * refer to significance at 1, 5, and 10%, respectively.

### Hypothesis Testing

#### Results of Principal Regression and Analysis

Time and industry fixed effects of unbalanced panel data were employed and all samples in Period *t* and Period *t*-1 were involved. The regression coefficients of Send_ratio are emphasized as they reflect the effects of distribution of stock dividends on enterprise innovations. The regression results are shown in Table 4. Therefore, H1 is verified.

**TABLE 4:** Results of principal regression.

Variable	Dependent variable: RD	
	(1) Current period (Period *t*)	(2) The period behind the current period (Period *t*-1)
Send_ratio	−0.0074***	−0.0139***
	(−4.0262)	(−3.1981)
Lnsize	0.0201***	0.0258***
	(10.3250)	(6.0282)
Lev	−0.0292***	−0.0347**
	(−4.9910)	(−2.3247)
ROA	−0.0805***	−0.1111*
	(−3.2231)	(−1.8788)
Tobin Q	0.0041***	0.0046***
	(6.9521)	(4.0371)
LnAge	−0.0077***	−0.0157***
	(−3.4187)	(−3.1875)
CFO	0.0454***	0.0557**
	(4.8030)	(2.2537)
TOP1	−0.0238***	−0.0280**
	(−4.3802)	(−2.4444)
Capital	0.0214*	0.0110
	(1.7335)	(0.4213)
PPETA	−0.0332***	−0.0374**
	(−5.3363)	(−2.2183)
Sales	−0.0194***	−0.0204***
	(−11.9286)	(−5.5514)
_cons	0.0206	–0.0669
	(0.9259)	(−1.3207)
Obs.	4313	1064
Adj.*R*^2^	0.425	0.400
Industry	YES	YES
Year	YES	YES

Numbers in the brackets are *t*-values, standard errors are clustered at the enterprise level, ***, **, * refer to significance at 1, 5, and 10%, respectively.

#### The Results of Examining the Effect of Equity Transfer on the Level of Innovation

According to the property right nature of enterprises, this article grouped regression tests the impact of equity transfer on the innovation level of enterprises. The regression results are shown in Table 5, which are in line with H2.

**TABLE 5:** Regression results of property right heterogeneity.

Variable	Dependent variable: RD			
	(1) Current period (Period *t*)		(2) The period behind the current period (Period *t*-1)	
	State-owned enterprises	Private enterprises	State-owned enterprises	Private enterprises
Send_ratio	0.0011	−0.0087***	–0.0105	−0.0133***
	(0.2365)	(−4.3797)	(−1.1925)	(−2.9518)
Lnsize	0.0158***	0.0211***	0.0357***	0.0236***
	(3.5212)	(9.6884)	(2.9026)	(5.5934)
Lev	−0.0368***	−0.0283***	–0.0140	−0.0431***
	(−2.6273)	(−4.5019)	(−0.3363)	(−3.0368)
ROA	–0.0768	−0.0840***	−0.2342*	–0.0993
	(−1.4779)	(−3.0417)	(−1.9780)	(−1.6064)
Tobin Q	0.0027**	0.0043***	0.0030	0.0048***
	(1.9745)	(6.8050)	(1.1076)	(3.9683)
LnAge	−0.0274***	−0.0057***	−0.0552***	−0.0113***
	(−3.1297)	(−2.6691)	(−3.1493)	(−2.5842)
CFO	0.0307	0.0492***	0.0479	0.0623**
	(1.4409)	(4.6673)	(0.7694)	(2.3059)
TOP1	−0.0293**	−0.0216***	–0.0347	−0.0219*
	(−2.3299)	(−3.6583)	(−1.1755)	(−1.9120)
Capital	0.0465	0.0227*	0.0405	0.0006
	(1.3248)	(1.6898)	(0.3936)	(0.0212)
PPETA	−0.0438***	−0.0288***	–0.0550	–0.0239
	(−3.8050)	(−3.8942)	(−1.6529)	(−1.4078)
Sales	−0.0151***	−0.0206***	−0.0304***	−0.0181***
	(−4.0312)	(−11.3996)	(−2.7088)	(−5.4641)
_cons	0.0675*	0.0165	–0.0011	–0.0837
	(1.7381)	(0.5962)	(−0.0113)	(−1.3493)
Obs.	822	3491	167	897
Adj.*R*^2^	0.464	0.428	0.483	0.396
Industry	YES	YES	YES	YES
Year	YES	YES	YES	YES

Numbers in the brackets are *t*-values, standard errors are clustered at the enterprise level, ***, **, * refer to significance at 1, 5, and 10%, respectively.

#### The Results of the Influence of Equity Transfer Firms With Different Ownership Concentration on the Level of Innovation

In this article, ownership concentration is measured by the shareholding ratio of the largest shareholder (TOP1), and the ownership concentration is divided into high and low groups according to industry-annual median. Table 6 shows regression results grouped according to ownership concentration, which is consistent with H3.

**TABLE 6:** Regression results of enterprises in Group A (high equity ownership concentration) and Group B (low equity ownership concentration).

Variable	Dependent variable: RD			
	(1) Current period (Period *t*)		(2) The period behind the current period (Period *t*-1)	
	High	Low	High	Low
Send_ratio	–0.028	−0.0117***	–0.0052	−0.0237***
	(−1.4392)	(−3.9999)	(−1.2775)	(−3.3397)
Lnsize	0.0197***	0.0204***	0.0285***	0.0249***
	(8.2626)	(6.9585)	(5.4374)	(4.0702)
Lev	−0.0236***	−0.0326***	–0.0218	−0.0407*
	(−3.6708)	(−3.4816)	(−1.2131)	(−1.7894)
ROA	−0.0792***	−0.0785*	–0.101	–0.0884
	(−2.7673)	(−1.9585)	(−1.3500)	(−1.0559)
Tobin Q	0.0036***	0.0045***	0.0043**	0.0046***
	(4.9851)	(5.0855)	(2.2713)	(3.1505)
LnAge	−0.0050**	−0.0106***	−0.0135***	−0.0205**
	(−2.0701)	(−2.8225)	(−2.7360)	(−2.3709)
CFO	0.0381***	0.0495***	0.0476*	0.0639*
	(3.0723)	(3.4814)	(1.8828)	(1.6575)
TOP1	–0.0090	–0.0211	0.0051	–0.0202
	(−0.9599)	(−1.5190)	(0.2470)	(−0.7278)
Capital	0.0096	0.0346*	–0.0228	0.0217
	(0.5614)	(1.8825)	(−0.6958)	(0.5539)
PPETA	−0.0253***	−0.0404***	–0.0013	−0.0767**
	(−3.6883)	(−3.9931)	(−0.0917)	(−2.5272)
Sales	−0.0193***	−0.0202***	−0.0234***	−0.0204***
	(−9.8732)	(−8.0891)	(−5.9023)	(−3.6009)
_cons	0.0023	0.0407	–0.102	–0.0513
	(0.0906)	(1.1513)	(−1.4582)	(−0.7386)
Obs.	2109	2204	533	531
Adj.*R*^2^	0.428	0.425	0.386	0.407
Industry	YES	YES	YES	YES
Year	YES	YES	YES	YES

Numbers in the brackets are *t*-values, standard errors are clustered at the enterprise level, ***, **, * refer to significance at 1, 5, and 10%, respectively.

#### From the Perspective of Industry Heterogeneity, the Impact of Equity Transfer on the Level of Innovation of Enterprises Is Investigated

According to (The Guidelines on Sector Classification of Listed Companies), we defined C27, C37, C39, and I — respectively, correspond to the pharmaceutical manufacturing industry, railway, shipbuilding, aerospace and other transportation equipment manufacturing, computer, communications and other electronic equipment manufacturing and information transmission, software and information technology service industries — as high-tech enterprises according to the industry code of the Guidance on Industry Classification of Listed Companies released by CSRC in 2012. The specific regression results are shown in Table 7, which are in line with H4.

**TABLE 7:** Regression results of enterprises in different industries.

Variable	Dependent variable: RD			
	(1) Current period (Period *t*)		(2) The period behind the current period (Period *t*-1)	
	High-tech industry	Non-high-tech industry	High-tech industry	Non-high-tech industry
Send_ratio	−0.0139***	−0.0036***	−0.0201**	−0.0065*
	(−3.1949)	(−2.7978)	(−2.4300)	(−1.8347)
Lnsize	0.0372***	0.0082***	0.0396***	0.0093***
	(7.5164)	(6.5431)	(4.6728)	(3.6577)
Lev	−0.0337**	−0.0195***	–0.0418	–0.0169
	(−2.3082)	(−4.0860)	(−1.4414)	(−1.2902)
ROA	−0.1666***	−0.0337*	–0.146	–0.0985
	(−2.8273)	(−1.6936)	(−1.3540)	(−1.4316)
Tobin Q	0.0063***	0.0018***	0.0068***	0.0027***
	(5.5600)	(3.7250)	(3.1994)	(2.6709)
LnAge	−0.0089*	−0.0052**	–0.0123	−0.0142**
	(−1.7421)	(−2.4891)	(−1.4536)	(−2.2942)
CFO	0.0926***	0.0203***	0.1046*	0.0299
	(3.5415)	(2.9340)	(1.8582)	(1.5758)
TOP1	−0.0497***	−0.0103**	−0.0561**	–0.0177
	(−3.6874)	(−2.4020)	(−2.2030)	(−1.6261)
Capital	0.0430	0.00520	0.0210	0.0102
	(1.2094)	(0.5466)	(0.3196)	(0.4263)
PPETA	−0.0522***	−0.0182***	–0.0428	–0.0275
	(−2.9013)	(−3.4161)	(−1.1463)	(−1.5606)
Sales	−0.0331***	−0.0110***	−0.0303***	−0.0104***
	(−7.5157)	(−8.8676)	(−3.9959)	(−3.9390)
_cons	–0.0241	0.0905***	–0.122	0.0749**
	(−0.4650)	(6.7747)	(−1.2501)	(2.5085)
Inter-group different	*p*-value = 0.000		*p*-value = 0.082	
Obs.	1398	2915	410	654
Adj.*R*^2^	0.363	0.377	0.297	0.360
Industry	YES	YES	YES	YES
Year	YES	YES	YES	YES

Numbers in the brackets are *t*-values, standard errors are clustered at the enterprise level, ***, **, * refer to significance at 1, 5, and 10%, respectively.

### Robustness Test

#### Transfer to the “Agency Problem” Exclusion

Through the analysis of the existing research, found that sending stock is listed companies in China to attract investors and compiled “fantasy stories,” shares in the name of “illusion” and “right to fill effect” attract, investors to buy a high turn sent shares of listed companies, as share prices rose, far more than the stock’s intrinsic value, the inside and outside investors of listed companies (Bushman and Piotroski, 2006; Kumar, 2009; Weld et al., 2009; Chen et al., 2013; Devos et al., 2015) take this opportunity to “roll with the current” and reduce their holdings. Due to information asymmetry, small and medium investors do not know that the transfer of shares is a story compiled by the listed company to reduce its holdings. The research confirms that the transfer of shares is a way for the large shareholders to hollow out the small and medium shareholders, which is a manifestation of the agency problem.

As far as this article is concerned, it is necessary to further explore whether the transfer of shares affects enterprise innovation through the agency problem rather than the liquidity factor. In order to exclude the effect of the transfer of shares on enterprise innovation through agency problems, first of all, we try to include factors affecting corporate governance in the regression. After the inclusion of corporate governance factors, if there is still a significant negative number between the transfer of shares and the enterprise innovation level, then the agency problem in the transfer of shares can be excluded. Corporate governance variables included in our regression include: (1) Equity structure: separation rate of two rights (SEPER): separation degree of cash flow rights and control rights; Equity checks and balances (EGUI): the ratio of the sum of the shareholding ratios of the second to the tenth largest shareholders to the shareholding ratio of the largest shareholder; Institutional shareholding ratio (INSTIHOLD): The sum of shareholding ratios of various types of institutional investors. (2) Board of directors: Board size (LNBoard): the natural logarithm of the number of directors; Independent directors ratio (INDEP); Duality: whether the chairman of the board and the general manager have a combination of positions. (3) Incentive mechanism: executive shareholding ratio (MSR): the proportion of listed company’s shares held by senior executives; LN Salary: The natural logarithm of the top three executive salaries. (4) External supervision: whether the Big Four audits (BIG4); Degree of market competition (HHI2). Table 8 shows the empirical results after adding corporate governance variables:

**TABLE 8:** Stock dividends vs. enterprise innovations: elimination of agency issues.

Variable	Dependent variable: RD	
	(1) Current period (Period *t*)	(2)The period behind the current period (Period *t*-1)
Send_ratio	−0.0065***	−0.0110***
	(−3.7561)	(−2.7909)
Lnsize	0.0174***	0.0238***
	(8.6541)	(5.7385)
Lev	−0.0292***	−0.0455***
	(−5.1405)	(−3.3565)
ROA	−0.0922***	−0.1431**
	(−3.5488)	(−2.3217)
Tobin Q	0.0038***	0.0051***
	(6.4185)	(4.4276)
LnAge	−0.0075***	−0.0125***
	(−3.6802)	(−2.9943)
CFO	0.0371***	0.0502*
	(3.8359)	(1.8906)
TOP1	−0.0356***	−0.0414**
	(−4.0161)	(−2.3500)
Capital	0.0104	0.0047
	(0.8478)	(0.1631)
PPETA	−0.0284***	−0.0310*
	(−4.5765)	(−1.9181)
Sales	−0.0201***	−0.0208***
	(−12.3043)	(−6.6039)
SEPER	0.0001	0.0002
	(1.6322)	(0.8591)
EGUI	−0.0031**	–0.0035
	(−2.2050)	(−1.2781)
INSTIHOLD	0.0011	–0.0077
	(0.3283)	(−0.8296)
LNBoard	0.0051	0.0114
	(0.8790)	(0.9767)
INDEP	0.0259	0.0503
	(1.4596)	(1.3178)
Duality	0.0013	0.0038
	(0.7901)	(1.1098)
MSR	0.0000	0.0000
	(0.5310)	(−0.3988)
LnSalary	0.0108***	0.0162***
	(8.6968)	(6.1041)
BIG4	0.0020	0.0034
	(0.4112)	(0.2756)
HHI2	0.0178	0.0157
	(0.9228)	(0.5630)
_cons	−0.0764**	−0.2950***
	(−2.3800)	(−3.9874)
Obs.	3833	911
Adj.*R*^2^	0.456	0.456
Industry	YES	YES
Year	YES	YES

Numbers in the brackets are *t*-values, standard errors are clustered at the enterprise level, ***, **, * refer to significance at 1, 5, and 10%, respectively.

The regression results show that after the incorporation of corporate governance variables, there is still a significant negative correlation between the Send_ratio of the current period (t) and the lagging period (*t*-1) on the innovation level (RD) of the firm, indicating that the influence of the Send_ratio on the innovation level of the firm is through the liquidity factor rather than the agency problem.

To make the results convincing, possible agency issues were eliminated. First, Send_ratio and the operation parameters mentioned above were used as was used as the dependent and independent variables, respectively. The regression residual (Res) was used as the independent variable in Step 2 to eliminate possible agency issues. Second, regression was conducted with Res and RD as independent and dependent variables, respectively. The regression results shown in Table 9 remained stable after elimination of possible agency issues.

**TABLE 9:** Residual regression: elimination of agency.

Variable	Dependent variable: RD	
	(1) Current period (Period *t*)	(2) The period behind the current period (Period *t*-1)
Res	−0.0066***	−0.0115***
	(−3.6038)	(−2.6547)
Lnsize	0.0193***	0.0262***
	(9.8170)	(6.8489)
Lev	−0.0296***	−0.0429***
	(−5.1157)	(−3.0193)
ROA	−0.0677***	–0.0966
	(−2.6276)	(−1.5738)
Tobin Q	0.0039***	0.0050***
	(6.6154)	(4.1214)
LnAge	−0.0072***	−0.0127***
	(−3.4353)	(−2.9006)
CFO	0.0463***	0.0683**
	(4.6320)	(2.4867)
TOP1	−0.0193***	−0.0243**
	(−3.5118)	(−2.1386)
Capital	0.0136	–0.0021
	(1.0913)	(−0.0745)
PPETA	−0.0290***	–0.0240
	(−4.6651)	(−1.5287)
Sales	−0.0185***	−0.0190***
	(−11.5229)	(−6.2832)
_cons	0.0098	−0.1365**
	(0.4067)	(−2.4488)
Obs.	3833	911
Adj.*R*^2^	0.435	0.421
Industry	YES	YES
Year	YES	YES

Numbers in the brackets are *t*-values, standard errors are clustered at the enterprise level, ***, **, * refer to significance at 1, 5, and 10%, respectively.

#### Endogeneity Control

Endogeneity of stock dividends and enterprise innovations may be present. First, listed companies distributing stock dividends tend to have low innovation levels, resulting in omission and deviation induced by the sieving effect. Second, distribution of stock dividends itself may cause the differences observed. In other words, self-selection of samples may be present. Third, it is possible that innovation serves as the cause and distribution of stock dividends serves as the effect. Specifically, listed companies with low innovation levels tend to distribute stock dividends in order to enhance stock liquidity. Based on that, the effects of endogeneity on results of this study were controlled using propensity score matching (PSM), Heckman two-stage model and propensity score matching – difference in differences (DID-PSM) model, respectively.

##### Propensity score matching

Enterprises were divided into the Sent group and the control group according to distribution of stock dividends in the current year (yes = Sent group, no = control group). The logit model was employed for regression to calculate propensity and control variables in principal regression were involved for PSM. Table 10 shows sample features before and after matching. As observed, most variables of the Sent group and the control group were significantly different before matching, but not after matching. Hence, influences of other unmeasurable variables on the results can be excluded. Table 11 summarizes regression results of samples after matching. As observed, the correlation of stock dividends with enterprise innovations was significantly negative at 1%, even with potential missing variables considered. Hence, the conclusion of this study is solid.

**TABLE 10:** Features of samples before and after matching.

Variable	Unmatched	Mean			% reduction	*t*-test		V(T)/V(C)
	Matched	Treated	Control	% bias	|Bias|	*t*	*p* > |t|	
Lnsize	U	21.571	22.107	–45.7		–25.09	0.000	0.68*
	M	21.572	21.587	–1.3	97.2	–0.66	0.551	0.97
Lev	U	0.3232	0.42085	–50.31		–28.19	0.000	0.81*
	M	0.32348	0.32398	0.6	99.5	–0.13	0.899	0.97
ROA	U	0.06588	0.06719	60.5		32.57	0.000	0.58*
	M	0.06569	0.06736	–3.1	94.9	–1.54	–1.54	0.72*
Tobin Q	U	3.1358	2.135	49.8		30.01	0.000	1.38*
	M	3.1263	3.116	0.5	99.0	0.22	0.829	0.90*
LnAge	U	2.5745	2.7428	–40.8		–24.38	0.000	1.30*
	M	2.5761	2.5904	–3.5	91.5	–1.50	0.134	1.30*
CFO	U	0.045	0.04177	4.8		2.77	0.006	1.09*
	M	0.0449	0.04658	–2.5	48.0	–1.09	0.277	1.09*
TOP1	U	0.34711	0.34946	–1.6		–13.1	0.355	0.84*
	M	0.34692	0.34957	–1.9	–13.1	–0.87	0.382	0.89*
Capital	U	0.06265	0.05114	23.3		13.96	0.000	1.31*
	M	0.06252	0.06277	–0.5	97.8	–0.22	0.829	0.95
PPETA	U	0.17568	0.22148	–33.0		–18.13	0.000	0.68*
	M	0.17583	0.17666	–0.6	98.2	–0.31	0.756	1.05
Sales	U	20.904	21.475	–42.6		–23.59	0.000	0.74*
	M	20.906	20.922	–1.2	97.2	–0.59	0.555	0.99

The symbol * represents that there are still some differences in the variance of covariables between the two groups.

**TABLE 11:** Regression results of samples after matching.

Variable	Dependent variable: RD	
	(1) Current period (Period *t*)	(2) The period behind the current period (Period *t*-1)
Send_ratio	−0.0070***	−0.0144***
	(−3.6634)	(−2.8837)
Lnsize	0.0196***	0.0257***
	(10.2554)	(5.6116)
Lev	−0.0302***	−0.0344**
	(−5.3092)	(−2.0602)
ROA	−0.0791***	–0.100
	(−2.8610)	(−1.4078)
Tobin Q	0.0040***	0.0046***
	(6.3776)	(3.4688)
LnAge	−0.0079***	−0.0161***
	(−3.3015)	(−2.6929)
CFO	0.0459***	0.0458*
	(4.8875)	(1.6651)
TOP1	−0.0202***	−0.0234*
	(−3.7170)	(−1.9202)
Capital	0.0148	–0.0163
	(1.2206)	(−0.5970)
PPETA	−0.0318***	−0.0362**
	(−5.3535)	(−2.0427)
Sales	−0.0188***	−0.0205***
	(−12.1123)	(−5.3128)
_cons	0.0148	–0.0569
	(0.6700)	(−1.0243)
Obs.	3931	848
Adj.*R*^2^	0.430	0.391
Industry	YES	YES
Year	YES	YES

Numbers in the brackets are *t*-values, standard errors are clustered at the enterprise level, ***, **, * refer to significance at 1, 5, and 10%, respectively.

##### Heckman two-stage model

The Heckman two-stage model was employed to tackle possible self-selection issues of samples in studies of correlation of stock dividends with innovation. In Stage I, Probit estimation was applied for distribution of stock dividends (S) to predict its probability; the inverse Mills ratio (IMR) was calculated. In Stage II, IMR was introduced into the equation of regression of stock dividends on innovations as a new control variable to enhance the accuracy of regression results obtained. According to the nature of Heckman two-stage model, at least one variable that affects stock dividends but not innovation shall be introduced in Stage II. In this study, transfer capability (SZNL) was introduced. Table 12 shows the regression results of Heckman two-stage model. As observed, the regression results after introduction of IMR were significantly negative at 1%, demonstrating that the conclusion of this study is solid.

**TABLE 12:** Regression results of Heckman two-stage model.

Variable	Dependent variable: S (yes or no)	Dependent variable: RD	Dependent variable: S (yes or no)	Dependent variable: RD
	Current period (Period *t*)		The period behind the current period (Period *t*-1)	
	Stage I	Stage II	Stage I	Stage II
Send_ratio		−0.0103***		−0.0227***
		(−4.6244)		(−3.5415)
IMR		−0.0152***		–0.0142
		(−2.8152)		(−0.9556)
Lnsize	–0.0499	0.0201***	−0.1542***	0.0268***
	(−1.6045)	(8.6702)	(−4.2206)	(3.3037)
Lev	0.2391**	−0.0380***	0.4139***	−0.0474*
	(2.2332)	(−5.2275)	(3.3765)	(−1.9506)
ROA	6.4752***	−0.1948***	4.1723***	–0.132
	(15.3317)	(−4.1928)	(8.8297)	(−1.4568)
Tobin Q	0.0324***	0.0042***	0.0130	0.0025**
	(3.2246)	(5.6522)	(1.1466)	(2.0931)
LnAge	−0.1815***	−0.0092***	−0.1597***	−0.0170**
	(−4.6866)	(−2.9699)	(−3.6994)	(−2.1480)
CFO	−0.9699***	0.0700***	0.413	0.0178
	(−3.8908)	(5.3300)	(1.4499)	(0.4123)
TOP1	−0.2850***	−0.0184***	0.0876	−0.0340**
	(−2.8411)	(−2.7063)	(0.7723)	(−2.0131)
Capital	2.1534***	0.00230	1.1134***	0.0588
	(7.1242)	(0.1170)	(3.2679)	(1.2932)
PPETA	−0.4343***	−0.0292***	–0.177	−0.0448*
	(−3.4593)	(−3.8401)	(−1.2507)	(−1.7961)
Sales	−0.0694**	−0.0173***	–0.0520	−0.0215***
	(−2.4766)	(−9.4644)	(−1.6097)	(−3.0606)
SZNL	0.0915***		0.0836***	
	(16.2725)		(12.8542)	
_cons	1.7655***	0.0431	3.2243***	–0.0174
	(4.4673)	(1.6418)	(6.9896)	(−0.2504)
Obs.	12269	2636	9575	383
Adj.*R*^2^/Pseudo R2	0.1222	0.444	0.0934	0.449
Industry	YES	YES	YES	YES
Year	YES	YES	YES	YES

Numbers in the brackets are *t*-values, standard errors are clustered at the enterprise level, ***, **, * refer to significance at 1, 5, and 10%, respectively.

##### Difference in differences-propensity score matching

As discussed above, distribution of stock dividends leads to enhanced stock liquidity, thus increased pressure of hostile takeovers. As a result, those enterprises have to focus on short-term revenue at the cost of long-term development. Consequently, enterprise innovations degrade. In 2015, the control of the Vanke Group was attacked by external investors and this incident attracted great attention. In September 2016, the Administrative Measures for Major Assets Reorganization of Listed Companies was revised to reduce the space for stock price speculation and maintain the stability of financial market. After that, many listed companies amended articles of association to prevent hostile takeover. Therefore, the negative impacts of stock dividends on enterprise innovations would be relieved after 2016 as the pressure of hostile takeover is significantly reduced. The following model is established:


(2) $$R\,D_{\text{it}}=\beta_{0}+\beta_{1}\,T\,r\,e\,a\,t_{i}\times P\,o\,s\,t_{t}+\beta_{k}\,C\,V_{i}+Y\,e\,a\,r+I\,n\,d+\varepsilon \,i$$

Enterprises were divided into three groups according to industry-year varying rate of stock dividends (Jiang et al., 2017). The group with highest varying rate of stock dividends (treat) was assigned 1; the group with lowest varying rate of stock dividends (control) was assigned 0. The Post group was assigned 0 and 1 before and after, respectively. Herein, we focused on the treat × post regression coefficient. The regression results shown in Table 13 indicated that the treat × post regression coefficient was significantly positive at 5, suggesting that listed companies were exposed to reduced pressure of hostile takeovers and was more willing to invest in innovations after 2016. Additionally, it verified that stock dividends affect enterprise innovations by tuning stock liquidity.

**TABLE 13:** DID-PSM regression results.

Variable	Dependent variable: RD	
	(1) Current period (Period *t*)	(2) The period behind the current period (Period *t*-1)
Treat × Post	0.0130**	0.0466**
	(2.0872)	(2.0998)
Lnsize	0.0298***	0.0348***
	(6.1058)	(3.9268)
Lev	−0.0528**	−0.1246*
	(−2.5341)	(−1.7536)
ROA	−0.1381**	–0.349
	(−2.1264)	(−1.6488)
Tobin Q	0.0025**	–0.000900
	(2.1471)	(−0.3706)
LnAge	−0.0188***	−0.0341**
	(−3.0020)	(−2.1179)
CFO	0.0650***	–0.0282
	(2.7351)	(−0.3394)
TOP1	–0.0157	–0.0100
	(−1.1169)	(−0.2554)
Capital	0.0678**	0.151
	(2.0764)	(1.6317)
PPETA	−0.0443**	−0.1055*
	(−2.3039)	(−1.9330)
Sales	−0.0245***	–0.0106
	(−5.5960)	(−1.0355)
_cons	–0.0718	−0.3131*
	(−1.1766)	(−1.8059)
Obs.	717	132
Adj.*R*^2^	0.396	0.392
Industry	YES	YES
Year	YES	YES

Numbers in the brackets are *t*-values, standard errors are clustered at the enterprise level, ***, **, * refer to significance at 1, 5, and 10%, respectively.

##### Other robustness tests

To make the results convincing, robustness tests were conducted. First, variables were defined in different ways: RD was measured by R&D investment deflated by total. Second, R&D investment is replaced by 0 if it is missing. Third, sampling interval is changed: the regression starts from 2007. Fourth, two-way clustering of time and industry was involved. The regression results shown in Table 14 revealed that the correlation of stock dividends with enterprise innovations remained significantly negative. In other words, the proposed conclusion is solid.

**TABLE 14:** Regression results of robustness test.

Variable	(1) RD deflated by total assets		(2) Missing R&D investment is replaced by 0		(3) Sampling interval is changed		(4) Two-way clustering	
	Period *t*	Period *t*-1	Period *t*	Period *t*-1	Period *t*	Period *t*-1	Period *t*	Period *t*-1
Send_ratio	−0.0033***	−0.0053**	−0.0033***	−0.0060***	−0.0064***	−0.0139***	−0.0074***	−0.0139***
	(−3.5753)	(−2.1518)	(−3.9823)	(−2.7413)	(−3.874)	(−3.5502)	(−4.7197)	(−3.5172)
Lnsize	−0.0094***	−0.0080***	−0.0071***	−0.0063***	0.0150***	0.0202***	0.0201***	0.0258***
	(−10.8821)	(−3.7037)	(−9.4962)	(−3.4151)	(9.2951)	(5.6959)	(7.7544)	(6.6681)
Lev	−0.0051*	−0.0146*	−0.0048**	−0.0114*	−0.0305***	−0.0322**	−0.0292***	−0.0347**
	(−1.8483)	(−1.9321)	(−2.0402)	(−1.7686)	(−5.985)	(−2.5657)	(−4.2034)	(−2.1393)
ROA	0.0030	–0.0323	0.0086	–0.0132	−0.0796***	−0.0900*	−0.0805*	−0.1111**
	(0.2488)	(−1.0796)	(0.8103)	(−0.5436)	(−3.597)	(−1.8338)	(−1.9510)	(−2.0441)
Tobin Q	0.0017***	0.0018***	0.0015***	0.0015***	0.0038***	0.0040***	0.0041***	0.0046***
	(5.8366)	(3.1917)	(5.8649)	(3.0294)	(7.2361)	(3.8790)	(3.5725)	(4.8527)
LnAge	−0.0026***	−0.0050**	−0.0024***	−0.0037*	−0.0080***	−0.0138***	−0.0077***	−0.0157***
	(−2.6449)	(−2.2253)	(−2.7298)	(−1.8484)	(−4.0839)	(−3.3335)	(−3.4164)	(−2.9644)
CFO	0.0163***	0.0303***	0.0116***	0.0219**	0.0372***	0.0463**	0.0454***	0.0557**
	(3.5695)	(2.5919)	(3.1716)	(2.4464)	(4.8823)	(2.4352)	(3.2866)	(2.2235)
TOP1	−0.0104***	−0.0145***	−0.0090***	−0.0120***	−0.0214***	−0.0257***	−0.0238***	−0.0280***
	(−4.2164)	(−2.8376)	(−4.1899)	(−2.6566)	(−4.5502)	(−2.6014)	(−5.5992)	(−2.9080)
Capital	0.0186***	–0.00520	0.0156***	–0.00100	0.0167	0.0174	0.0214*	0.0110
	(3.3906)	(−0.4316)	(3.3581)	(−0.1016)	(1.5764)	(0.8311)	(1.6880)	(0.3817)
PPETA	−0.0160***	−0.0180**	−0.0140***	−0.0186***	−0.0303***	−0.0354**	−0.0332***	−0.0374***
	(−5.4040)	(−2.2627)	(−5.7120)	(−2.7775)	(−6.0016)	(−2.5320)	(−5.0650)	(−2.5960)
Sales	0.0091***	0.0101***	0.0067***	0.0078***	−0.0147***	−0.0167***	−0.0194***	−0.0204***
	(11.7021)	(5.0629)	(10.3671)	(4.6948)	(−10.8601)	(−5.4993)	(−10.9657)	(−6.9903)
_cons	0.0264**	–0.0092	0.0223**	–0.0111	0.0250	–0.0454	0.0206	–0.0669
	(2.0649)	(−0.2857)	(2.1217)	(−0.3996)	(1.3784)	(−1.0888)	(0.8519)	(−1.4734)
Obs.	4313	1064	4979	1230	4979	1230	4313	1064
Adj.*R*^2^	0.351	0.310	0.408	0.378	0.460	0.445	0.425	0.400
Industry	YES	YES	YES	YES	YES	YES	YES	YES
Year	YES	YES	YES	YES	YES	YES	YES	YES

Numbers in the brackets are *t*-values, standard errors are clustered at the enterprise level, ***, **, * refer to significance at 1, 5, and 10%, respectively.

#### Further Testing: Mechanism Analysis

As discussed above, stock dividends have significant negative impacts on enterprise innovations. This can be theoretically attributed to the fact that distribution of stock dividends leads to increased stock liquidity, thus increased risk of hostile takeover. In this section, this proposal is verified by investigate the mediating effect of stock liquidity on the effects of stock dividends on enterprise innovations. Based on conventional procedures of mediating effect testing, we establish the following models:


(3) $$L\,i\,q\,u\,i\,d\,i\,t\,y_{i}=\beta_{0}+\beta_{1}\,S\,e\,n\,d\,\_\,r\,a\,t\,i\,o_{i}+\beta_{k}\,C\,V_{i}+Y\,e\,a\,r+I\,n\,d+\varepsilon \,i$$

(4) $$R\,D_{i}=\beta_{0}+\beta_{1}\,S\,e\,n\,d\,\_\,r\,a\,t\,i\,o_{i}+\beta_{1}\,L\,i\,q\,u\,i\,d\,i\,t\,y+\beta_{k}\,C\,V_{i}+Y\,e\,a\,r+I\,n\,d+\varepsilon \,i$$

The stock liquidity was reflected by the turnover rate (Menkveld and Wang, 2013; Egginton, 2014), with other variables remained unchanged. The regression results shown in Table 15 revealed the correlation of Send_ratio with RD in Period *t*. The regression coefficient of stock dividends on stock liquidity was insignificantly positive (Line 1); with both stock dividends and stock liquidity included in regression, coefficients of stock dividends and liquidity were significantly negative (Line 2). Sobel test revealed that *P* = 0.00, demonstrating the presence of mediating effect. In other words, stock dividends decrease enterprise innovations by increasing stock liquidity. This is consistent with previous studies (Fang et al., 2014).

**TABLE 15:** Mechanism verification.

Variable	Period *t*		Period *t*-1	
	(1) Dependent variable: liquidity	(2) Dependent variable: RD	(1) Dependent variable: liquidity	(2) Dependent variable: RD
Send_ratio	0.357	−0.0077***	1.1157***	−0.0126**
	(1.5535)	(−3.5859)	(2.5976)	(−2.5516)
Liquidity		−0.0003**		–0.0000
		(−2.2191)		(−0.0648)
Lnsize	−2.7108***	0.0212***	−2.7361***	0.0256***
	(−11.3636)	(8.5504)	(−6.4555)	(5.2988)
Lev	1.028	−0.0282***	–0.760	–0.0246
	(1.3919)	(−4.0084)	(−0.5294)	(−1.4949)
ROA	−6.4954**	−0.1104***	–6.847	−0.1255*
	(−2.0419)	(−3.6704)	(−1.2508)	(−1.8566)
Tobin Q	0.3326***	0.0042***	−0.2601***	0.0047***
	(4.6662)	(6.4568)	(−2.8725)	(3.8307)
LnAge	0.4133*	−0.0080***	0.177	−0.0184***
	(1.8557)	(−3.0131)	(0.4498)	(−3.3560)
CFO	−9.9792***	0.0583***	−7.7144***	0.0677**
	(−6.3880)	(5.2332)	(−2.9922)	(2.2660)
TOP1	1.1678*	−0.0246***	1.292	−0.0272**
	(1.6998)	(−3.7521)	(1.0930)	(−2.2225)
Capital	12.6579***	0.0302**	0.882	0.0139
	(7.1266)	(2.0339)	(0.3013)	(0.4625)
PPETA	–0.805	−0.0402***	–2.431	−0.0380*
	(−0.8530)	(−4.9675)	(−1.4120)	(−1.8240)
Sales	0.4855**	−0.0214***	0.250	−0.0217***
	(2.2847)	(−10.5770)	(0.6657)	(−5.0557)
_cons	55.1550***	0.0422	57.4366***	–0.0342
	(18.9967)	(1.4428)	(10.2027)	(−0.6278)
Obs.	3368	3368	894	894
Adj.*R*^2^	0.393	0.423	0.440	0.391
Industry	YES	YES	YES	YES
Year	YES	YES	YES	YES

Numbers in the brackets are *t*-values, standard errors are clustered at the enterprise level, ***, **, * refer to significance at 1, 5, and 10%, respectively.

Next, the correlation of Send_ratio with RD in Period *t*-1 was investigated. The regression coefficient of stock dividends on stock liquidity was significantly positive (Line 1), even at 1%, demonstrating that stock dividends lead to enhanced stock liquidity; with both stock dividends and stock liquidity included in regression, coefficient of stock dividends was significantly negative, while that of stock liquidity was insignificantly negative. Sobel test revealed that *P* = 0.66, demonstrating the absence of mediating effect. This may be attributed to the fact that innovation decision-making of enterprises will be interfered by other factors in the ever-changing market. Nevertheless, The regression coefficient of stock dividends on stock liquidity was significantly positive in Period *t*-1, even at 1%.

## Discussion

On the basis of social psychology theory, from the perspective of management short-term psychological preference and irrational investor psychology, and from the perspective of stock liquidity, this article uses the data of China’s A-share listed companies to empirically investigate the impact of high transfer policy on enterprise innovation, and finds that: High transfer dividend is mainly through improving stock liquidity, which makes the company face greater risk of hostile merger and acquisition, thus making the management have psychological preference to focus on short-term effect and reduce the level of enterprise R&D and innovation. The details are as follows.

First, the degree of stock transfer and the level of enterprise innovation have a significant negative effect.

In Table 4, the regression results of *T* period and *T*-1 period show that the regression coefficients of Send_ratio are −0.0074 and −0.0139, respectively, both of which are significantly negative correlated at the level of 1%, indicating that equity conversion signifies the innovation level of enterprises. The results were compared with those of Ferreira et al. (2012); Fang et al. (2014), and Pokorna and Sebestova (2019). On the one hand, the information asymmetry between the management and the shareholders causes the managers to pay attention to the short-term benefits of the business performance of the enterprise, which makes them sacrifice the long-term innovation activities in exchange for the current profits, so as to avoid the stock undervaluation. On the other hand, high liquidity reduces the cost of capital in and out of the enterprise, which increases the possibility that the enterprise is faced with hostile merger and acquisition, and the management will face the risk of unemployment and reputation loss for failing to run the enterprise well (da Costa and Mata, 2016). Therefore, the management will pay more attention to short-term performance and the level of stock price. It shows that under the dual pressure of performance pressure and the increasing possibility of acquisition, the management tends to be conservative and unwilling to carry out risky innovation activities under the combined action of internal motivation and extrinsic motivation.

Second, compared with state-owned enterprises, the innovation activities of private enterprises are more affected by equity transfer.

After property rights division, whether the Send_ratio test of *T* period or *T*-1 period affects the innovation level (RD), the grouping regression results of private enterprises are significantly negative correlated at the 1% level, while those of state-owned enterprises are not significant, indicating that the impact of equity transfer on the innovation level of private enterprises is more serious. According to the research results of Oliver et al. (1997); Jefferson et al. (2006), and Ni et al. (2019), the innovation efficiency of state-owned enterprises is lower than that of private enterprises, mainly because state-owned enterprises lack effective incentives to promote innovation and improve the operating efficiency and benefits of enterprises. Due to the lack of actual shareholders and the influence of the thought of “official standard,” the management of many state-owned enterprises do not care about increasing output and maximizing profits under the circumstance of relatively fixed salary and less pressure of being acquired, so their willingness to carry out innovative activities is bound to be low. The private sector must focus more on increasing productivity and maximizing profits (Ni et al., 2019). Therefore, private enterprises are greatly affected by the transfer of shares and their innovation activities are greatly affected.

Third, compared with the enterprises with high ownership concentration, the innovation activities of the enterprises with low ownership concentration are more affected by the transfer of shares.

No matter in *T* period or *T*-1 period, the impact of Send_ratio on the firm’s innovation level (RD) is significantly negatively correlated in the low ownership concentration group. Consistent with the results of Stein (1988), the liquidity hostile takeover pressure caused by the transfer of shares forced major shareholders to gain short-term effects by sacrificing long-term projects. Major shareholders have more rights than those with lower ownership concentration, which aggravates the second type of agency problem. Major shareholders have the motivation to occupy minority shareholders through tunnel behavior, which leads to fewer innovation activities in enterprises with higher ownership concentration than those with lower ownership concentration (Xu et al., 2018). Similar to the results of the split ownership analysis, the enterprises with low ownership concentration are more affected by the transfer of shares and the innovation activities are more affected.

Fourth, the innovation activity of high and new technology industry is affected weakly by the transfer of shares.

According to the data of industry heterogeneity, the influence coefficient of firm innovation level (RD) in high-tech industry is smaller, and the difference between groups is significant. It shows that the effect of stock transfer on the innovation level of high-tech enterprises is weaker than that of non-high-tech enterprises. As stated by Hsu et al. (2014), high-tech enterprises are innovative enterprises engaged in high-risk investment. Innovation is the characteristic of high-tech enterprises. Whether an enterprise is a good high-tech enterprise depends not only on whether it continues to carry out innovation activities, but also on whether its innovation activities have the potential to lead the direction of the industry. Investors focus on the future of the enterprise (Hou et al., 2019). Therefore, even if the transfer of shares has a inhibiting effect on the innovation activities of high-tech enterprises, high-tech enterprises will still maintain continuous innovation, so the inhibiting effect is weaker than that of non-high-tech enterprises.

## Research Results

### Research Conclusion

Stock liquidity is an important factor affecting enterprise innovation. This article studies the effect of stock transfer on stock liquidity and then on enterprise innovation level. The results show that stock transfer reduces innovation input, and the relationship still exists after a series of endogenous and robustness tests. Further research shows that in private enterprises, ownership concentration and non-high-tech enterprises, the effect of equity transfer on reducing the level of innovation is more significant. Excluding the agency problem that may exist in the reduction of holdings due to “insiders,” the conclusion is still stable. At the same time, it is found that the effect of stock transfer on innovation level is through improving stock liquidity, and the liquidity increases the pressure of listed companies facing hostile takeover, and the management pays more attention to the short-term performance of enterprises, thus reducing the investment in innovation. The research results support the stock liquidity hypothesis of stock transfer.

The research conclusion of this article has important theoretical and practical significance. First, it provides a new perspective of enterprise innovation research. This article brings the transfer stock into the analytical framework of innovation and provides a new research perspective for exploring the influencing factors of innovation. Second, enrich the related theory of transferring stocks. In this article, we explore that stock transfer increases the risk of hostile merger and acquisition by increasing stock liquidity, thereby reducing the innovation behavior of enterprises, and verifying the liquidity hypothesis. Third, enriched the empirical evidence of the transfer. This article empirically tests the relevance of the transfer of shares to enterprise innovation, and analyzes the motivation of the transfer of shares, and further understands the economic significance and economic consequences of the transfer of shares.

### Implication

When faced with short-term pressure, the management of listed companies may sacrifice long-term development to achieve short-term performance. First of all, we should clearly understand the innovation behavior. We should face continuous innovation in a scientific and rational way, and realize that it is necessary and worthwhile to adhere to scientific and reasonable innovation input. Although it cannot directly improve the performance of enterprises in the short term, it will bring long-term economic benefits to enterprises. Second, build a long-term mechanism. Listed companies need to design a reasonable incentive system and establish long-term values, so that the management level can be based on their own professional reputation and career prospects, make earnest efforts for the company, encourage the management level to more actively undertake innovation risks, and reasonably guarantee the reasonable income of the management level, so as to promote the improvement of the innovation level of the enterprise. Third, we will deepen the reform of state-owned enterprises. Further improve the mode of development, and constantly stimulate the vitality of enterprises. Due to the lack of actual shareholders of state-owned enterprises and the inherent “official standard” thought of the management, the innovation willingness of the management is not strong, so it is necessary to carry out various ways of incentives and continuously improve the degree of marketization to fully stimulate the initiative of the management of state-owned enterprises. Fourth, optimize the ownership structure. The existence of major shareholders weakens the first type of agency problem in some respects, but it also brings the second type of agency problem because of the potential encroachment on the long-term interests of other shareholders. By effectively setting up the ownership structure of the company, the tunnel effect brought by the major shareholders can be restrained, and the major shareholders can effectively supervise and restrain the management to make decisions to enhance the value of the company by actively participating in the corporate governance. Finally, high and new technology industry should be vigorously developed. High and new technology can improve the innovation performance of enterprises, so as to provide vitality and benefits for the development of enterprises and the country. Therefore, policy subsidies and guidance should be provided to the high-tech industry continuously, and the development direction of enterprises should be reasonably allocated and guided, so as to improve the overall innovation efficiency.

Future research could mainly based on behavioral finance and social psychology, building a bridge between dividend policy and enterprise innovation from the perspective of management characteristics. On the one hand, in the face of the same market, how will enterprises affect the behavior of corporate management for dividend payments, especially for the stock dividends. On the other hand, how do the results of these behaviors influence the management to make decisions on enterprise development? Or how the characteristics of subordinates, such as gender and education, will affect the behavior of the company’s stock dividends.

## Data Availability Statement

Publicly available datasets were analyzed in this study. This data can be found here: https://www.gtarsc.com/.

## Author Contributions

ZL and HL designed and collected the data, analyzed the data, and wrote the manuscript. XK advised and supported in all the aforementioned tasks. All authors contributed to the article and approved the submitted version.

## Conflict of Interest

The authors declare that the research was conducted in the absence of any commercial or financial relationships that could be construed as a potential conflict of interest.

## Publisher’s Note

All claims expressed in this article are solely those of the authors and do not necessarily represent those of their affiliated organizations, or those of the publisher, the editors and the reviewers. Any product that may be evaluated in this article, or claim that may be made by its manufacturer, is not guaranteed or endorsed by the publisher.
